# Asthmatic lung fibroblasts promote type 2 immune responses *via* endoplasmic reticulum stress response dependent thymic stromal lymphopoietin secretion

**DOI:** 10.3389/fphys.2023.1064822

**Published:** 2023-01-25

**Authors:** Li Y. Drake, Maunick Lefin Koloko Ngassie, Benjamin B. Roos, Jacob J. Teske, Y. S. Prakash

**Affiliations:** ^1^ Department of Anesthesiology and Perioperative Medicine, Mayo Clinic, Rochester, MN, United States; ^2^ Department of Pathology and Medical Biology, University of Groningen, University Medical Center Groningen, Groningen, Netherlands; ^3^ Groningen Research Institute for Asthma and COPD, University of Groningen, University Medical Center Groningen, Groningen, Netherlands; ^4^ Department of Physiology and Biomedical Engineering, Mayo Clinic, Rochester, MN, United States

**Keywords:** asthma, lung fibroblast, TSLP, TNF, endoplasmic reticulum stress

## Abstract

Lung fibroblasts contribute to asthma pathology partly through modulation of the immune environment in the airway. Tumor necrosis factor-α (TNFα) expression is upregulated in asthmatic lungs. How asthmatic lung fibroblasts respond to TNFα stimulation and subsequently regulate immune responses is not well understood. Endoplasmic reticulum (ER) stress and unfolded protein responses (UPR) play important roles in asthma, but their functional roles are still under investigation. In this study, we investigated TNFα-induced cytokine production in primary lung fibroblasts from asthmatic vs. non-asthmatic human subjects, and downstream effects on type 2 immune responses. TNFα significantly upregulated IL-6, IL-8, C-C motif chemokine ligand 5 (CCL5), and thymic stromal lymphopoietin (TSLP) mRNA expression and protein secretion by lung fibroblasts. Asthmatic lung fibroblasts secreted higher levels of TSLP which promoted IL-33-induced IL-5 and IL-13 production by peripheral blood mononuclear cells. TNFα exposure enhanced expression of ER stress/UPR pathways in both asthmatic and non-asthmatic lung fibroblasts, especially inositol-requiring protein 1α in asthmatics. ER stress/UPR inhibitors decreased IL-6, CCL5, and TSLP protein secretion by asthmatic lung fibroblasts. Our data suggest that TNFα and lung fibroblasts form an important axis in asthmatic lungs to promote asthmatic inflammation that can be attenuated by inhibiting ER stress/UPR pathway.

## 1 Introduction

Lung fibroblasts play important roles in asthma by regulating asthmatic inflammation and airway tissue remodeling (Michalik et al., 2018). They regulate asthmatic inflammation by responding to and producing proinflammatory cytokines. Tumour necrosis factor (TNF)α is a pleiotropic proinflammatory cytokine involved in many aspects of asthma pathology (Brightling et al., 2008). TNFα expression is upregulated in asthmatic lungs, particularly in patients with severe refractory asthma or neutrophilic asthma (Ying et al., 1991; Bradding et al., 1994; Howarth et al., 2005; Berry et al., 2006; Brightling et al., 2008; Baines et al., 2011; Kuo et al., 2017). Increased sputum TNFα levels are associated with severe asthma exacerbations (Ghebre et al., 2019). Although anti-TNFα therapy has produced varied results in clinical trials, it has shown beneficial effects in subpopulations of asthma patients that warrant further understanding of the functional role of TNFα in asthma (Brightling et al., 2008; Malaviya et al., 2017). TNFα stimulates lung fibroblasts to secrete proinflammatory cytokines and chemokines, such as transforming growth factor beta (TGF-β), granulocyte-macrophage colony-stimulating factor (GM-CSF), monocyte chemoattractant protein-1 (MCP-1), eotaxin, interleukin 6 (IL-6), and IL-8 (Fitzgerald et al., 2003; Sabatini et al., 2003; Sullivan et al., 2005; Yap et al., 2020a). How asthma impacts TNFα-induced cytokine responses in human lung fibroblasts is not well characterized. Moreover, little is known regarding the downstream effects of the asthmatic fibroblast-derived cytokines on immune responses, or the mechanisms that link TNFα effects to immune responses.

The endoplasmic reticulum (ER) has multiple cellular functions including protein biosynthesis, folding, and post-translational modifications (Anelli and Sitia, 2008). ER stress occurs when ER homeostasis is impaired with accumulation of unfolded and/or misfolded proteins in the ER lumen (Kaufman, 2002). To restore ER homeostasis, cells develop an adaptive response termed the unfolded protein responses (UPR) (Ron and Walter, 2007; Smith, 2018). The signaling pathways in ER stress sensing and responses are well-established (Ron and Walter, 2007; Smith, 2018) (Junjappa et al., 2018) and involve three major sensors (inositol-requiring protein 1α (IRE1α), protein kinase RNA-like endoplasmic reticulum kinase (PERK), and activating transcription factor 6 (ATF6)). IRE1 is the most prominent and evolutionarily conserved, with two isoforms: IRE1α expressed by almost all tissues and IRE1β only by intestinal epithelial cells and airway mucous cells (Junjappa et al., 2018). Upon ER stress, IRE1α is activated by autophosphorylation and then cleaves 26 nucleotides out of the X-box binding protein 1 (XBP1) mRNA to generate spliced XBP1 (XBP1s), a transcription factor that regulates multiple genes. IRE1 can also degrade other mRNAs *via* regulated Ire1-dependent decay (RIDD). The relevance of ER stress/UPR lies in the fact that TNFα has been shown to activate this pathway in several cell types, including airway smooth muscle and bone marrow mesenchymal stem cells (Xue et al., 2005; Denis et al., 2010; Yap et al., 2020b; Zhao et al., 2021). Whether TNFα has a similar role in lung fibroblasts and whether asthma dysregulates the ER stress/UPR pathway in these cells is not known. In this study, we treated asthmatic and non-asthmatic primary human lung fibroblast with TNFα and then analyzed the ER stress/UPR pathway as a potential mechanism contributing to downstream cytokine responses. We found that asthmatic lung fibroblasts secrete more TSLP that subsequently promotes type 2 immune responses, and we identified the IRE1α pathway as a contributor to TNFα-induced cytokine secretion.

## 2 Material and methods

### 2.1 Fibroblast isolation and culture

Primary human lung fibroblasts were isolated from lung tissues from patients who had undergone thoracic surgery at St. Mary’s Hospital, Mayo Clinic Rochester, MN. These studies were approved by Mayo Clinic Institutional Review Boards. Patients were informed and consented by research coordinators during their clinic visits prior to surgical decisions, and samples were obtained only from those patients who provided a written or video/verbal consent followed by electronic signature for the use of their tissues and relevant medical records for research. Upon acquisition of tissues, relevant clinical data were recorded, and all patient identifiers were deleted, and the samples given unique numbers to provide anonymization. Clinical characteristics of patients are listed in Supplementary Table S1. A total of eight non-asthmatic and seven asthmatic patient samples were used. Asthma patients were identified by diagnostic criteria as listed in their medical records. The process for isolation and characterization of lung fibroblasts has been described in detail previously (You et al., 2019). Briefly, parenchymal lung tissue was dissected and enzymatically dissociated using 1 mg/ml of collagenase IV. Isolated cells were then sub-cultured under standard conditions at 37°C and 5% CO_2_ in Dulbecco’s minimum essential medium (DMEM) with 10% fetal bovine serum (FBS; R&D Systems, Minneapolis, MN) and 1% antibiotics/antimycotic. Primary lung fibroblasts were used between passages three and five for this study.

### 2.2 Reagents

Human TNFα (#210-TA) and IL- 33 (#3625-IL) were purchased from R&D Systems. Human IL-2 was purchased as Proleukin (aldesleukin) from Novartis Pharmaceuticals Corporation. Anti-TSLP antibody was purchased from R&D (AF1398). Antibodies recognizing Collagen I (ab34710) and Fibronectin (ab2413) were purchased from Abcam. Antibody recognizing Collagen IV was purchased from Novus Biologicals (NB120-6586SS). Tauroursodeoxycholic acid (TUDCA) was purchased from Sigma (T0266). All antibodies recognizing ER stress proteins were purchased from Cell Signaling, IRE1α, #3294; ATF-6, #65880; XBP1-S, #40435S; Binding immunoglobulin protein (BiP), #3177; PERK, #5683. Anti-phospho-IRE1α antibody was purchased from Novus Biologicals (NB100-2323). Anti-β-actin antibody was purchased from Sigma (A5316).

### 2.3 Fibroblast treatments

Lung fibroblasts were serum-starved with DMEM for 2 days and then were treated with 20 ng/ml TNFα for indicated time. In some experiments, fibroblasts were pre-treated with IRE1 inhibitor I at 25 µM final concentration or TUDCA at 500 μg/ml final concentration for 1 h prior to TNFα treatment. Cell lysates and culture supernatants were harvested and stored at ‒80°C until analyses.

### 2.4 Peripheral blood mononuclear cell (PBMC) culture

PBMCs were isolated using Histopaque 1,077 (Sigma-Aldrich) from the blood of anonymous healthy adult donors recruited at Mayo Clinic Blood Bank. PBMCs were washed and resuspended in RPMI 1640 medium (Gibco/Life Technologies) containing 10% heat-inactivated human AB serum (Sigma-Aldrich) and 1% antibiotics. Cells were cultured in 96-well round-bottom plates (3 × 10^5^ cells/well) with IL-2 (20 units/mL) and IL- 33 (50 ng/ml). Fibroblast-derived culture supernatants were added at 10% of the final volume in the PBMC culture. For some experiments, PBMCs were pretreated with anti-TSLP antibody (1 μg/ml) for 15 min before adding other stimuli. PBMC culture supernatants were harvested after 4 days. IL-5 and IL-13 concentrations in the cell-free supernatants were determined using ELISA kits.

### 2.5 Quantitative RT-PCR

Total RNA was extracted from cells. cDNA was synthesized using standard techniques. PCR was performed using a Roche LightCycler LC96. Ct values were normalized to housekeeping gene S16, and fold changes of gene expression relative to vehicle control non-asthmatic fibroblasts were calculated by the ΔΔCt method. Primers used were from IDT. The primer sequences are listed in Supplementary Table S2.

### 2.6 WES analysis

Protein expression was measured using WES (ProteinSimple, San Jose CA, United States), a capillary based immunoblotting system. Following the manufacturer’s instructions, 0.3 µg cellular protein was loaded into 12-230-kDa or 66-440-kDa WES separation modules with appropriate primary and secondary antibodies validated for WES system. Digital representations of the electropherograms were used and then quantified using Compass for Simple Western Software. All antibodies recognizing ER stress proteins were used at 1:50 dilution. Protein expression was normalized to β-actin expression (used at 1:100 dilution).

### 2.7 Immunofluorescence staining

Lung fibroblasts were grown in four well chamber slides to 70% confluence, serum starved for 2 days, and then treated with TNFα (20 ng/ml) for 24 h. Subsequently, fibroblasts were fixed with 4% paraformaldehyde, permeabilized, immunostained for phospho-IRE1α (antibody was used at 1:100) and a fluorescence-labeled secondary antibody using standard procedures. Nuclei were counterstained with DAPI. Two fields were randomly selected for each slide and imaged at ×20 magnification using a Keyence BZX-800 system. The fluorescence intensity in the selected fields were analyzed using the image analysis software QuPath. The results were presented as intensity per cell.

### 2.8 ELISA

Duoset ELISA kits (R&D Systems) were used to measure IL-5, IL-6, IL-8, IL-13, CCL5 and TSLP following the manufacturer’s protocol. Absorbance was read at 450 and 530 nm using the FlexStation (Molecular Devices, San Jose, CA). Standard curve was generated to calculate the cytokine concentrations in samples.

### 2.9 Statistical analyses

Data were analyzed using GraphPad Prism 8.2.0 (GraphPad, San Diego, CA). Outliers were determined using Grubbs’ test (α = 0.05) in GraphPad software and eliminated from further analysis. Statistical significance between groups was assessed using either paired or unpaired two-tailed Student’s t-tests as appropriate using GraphPad software. Values are expressed as mean ± standard deviation (SD) and *p*-value <0.05 was considered statistically significant.

## 3 Results

### 3.1 Asthmatic lung fibroblasts secrete more CCL5 at baseline but more TSLP after TNFα stimulation

**FIGURE 1:**
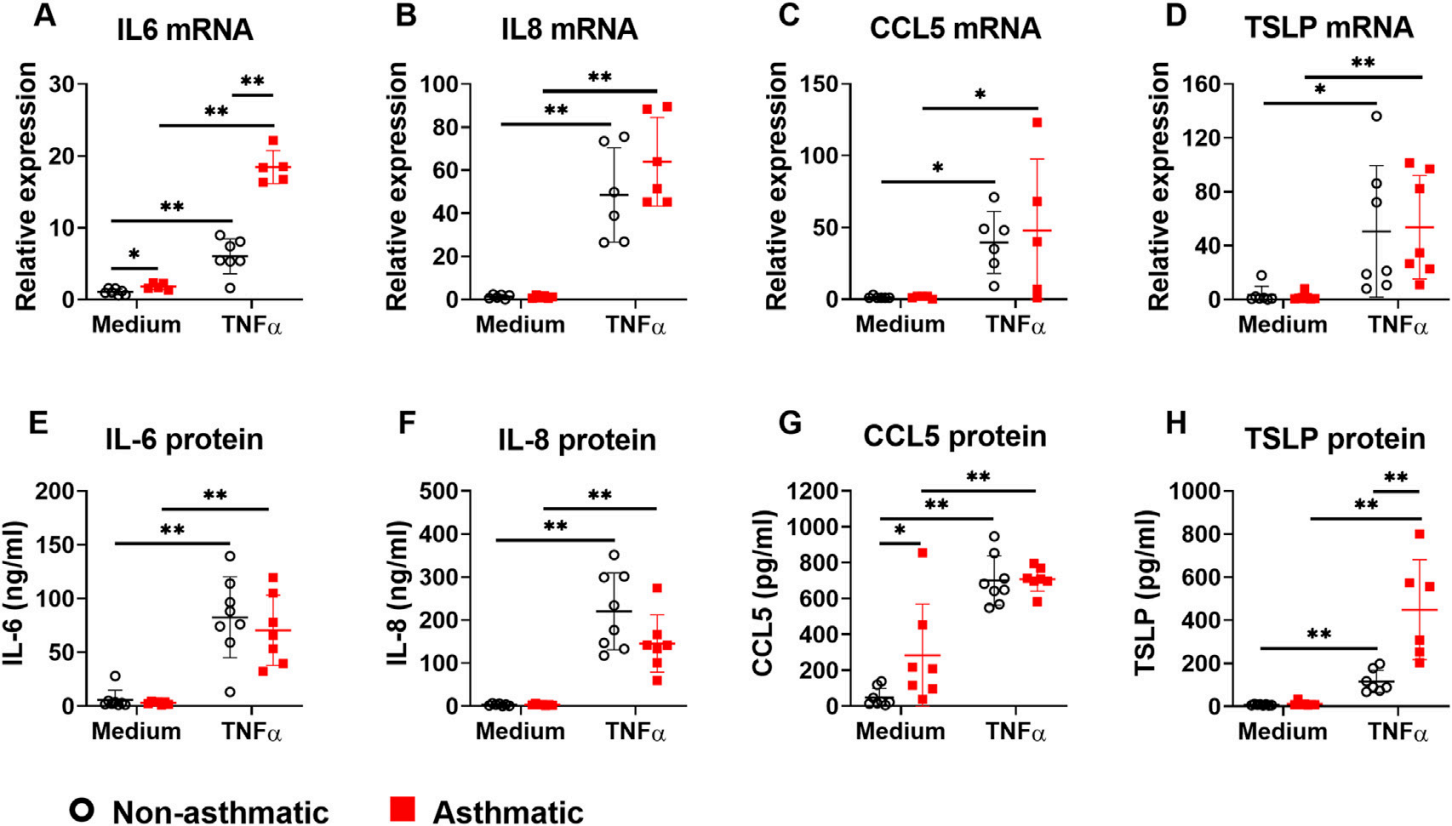
The expression of proinflammatory cytokines/chemokines in asthmatic and non-asthmatic human lung fibroblasts. Serum-starved fibroblasts were cultured with medium or TNFα (20 ng/ml) for 24 h or 3 days. RNA was extracted from the cells cultured for 24 h. Culture supernatants were harvested after 3-day culture. Gene expression was determined by quantitative PCR **(A–D)**. The levels of cytokines/chemokines in the culture supernatants were determined by ELISA **(E–H)**. Data are shown as mean ± SD from N = 5–9 patient samples. Each dot represents one patient sample. * indicates significant difference *p* < 0.05. ** indicates significant difference *p* < 0.01.

To investigate whether asthmatic human lung fibroblasts have enhanced cytokine responses that drive asthmatic inflammation, we stimulated lung fibroblasts from non-asthmatic and asthmatic human subjects with TNFα (20 ng/ml) for 24 h and then measured the mRNA expression of proinflammatory cytokines and chemokines that are critically involved in asthma, including IL-6, IL-8, CCL5, and TSLP. At baseline (medium control), only IL-6 mRNA expression was higher in asthmatic fibroblasts compared to non-asthmatic controls (Figures 1A–D). TNFα stimulation induced upregulation of IL-6, IL-8, CCL5, and TSLP mRNA expression in both asthmatic and non-asthmatic fibroblasts. TNFα-induced IL-6 mRNA levels were significantly higher in asthmatics compared to non-asthmatics whereas TNFα-induced IL-8, CCL5, and TSLP mRNA levels were comparable between these two groups.

Next, we examined TNFα-induced cytokine secretion by asthmatic and non-asthmatic lung fibroblasts. At baseline, asthmatic fibroblasts secreted significantly higher levels of CCL5 than non-asthmatic fibroblasts; IL-6, IL-8, and TSLP levels were not significantly different between asthmatics and non-asthmatics (Figures 1E–H). TNFα treatment increased secretion of IL-6, IL-8, CCL5, and TSLP in both non-asthmatic and asthmatic fibroblasts. There were no significant differences between asthmatic and non-asthmatic in IL-6, IL-8, and CCL5 secretion. However, TSLP secretion by asthmatic fibroblasts was significantly higher than non-asthmatic controls (Figures 1E–H).

### 3.2 TNFα stimulation does not increase ECM deposition by asthmatic lung fibroblasts

**FIGURE 2:**
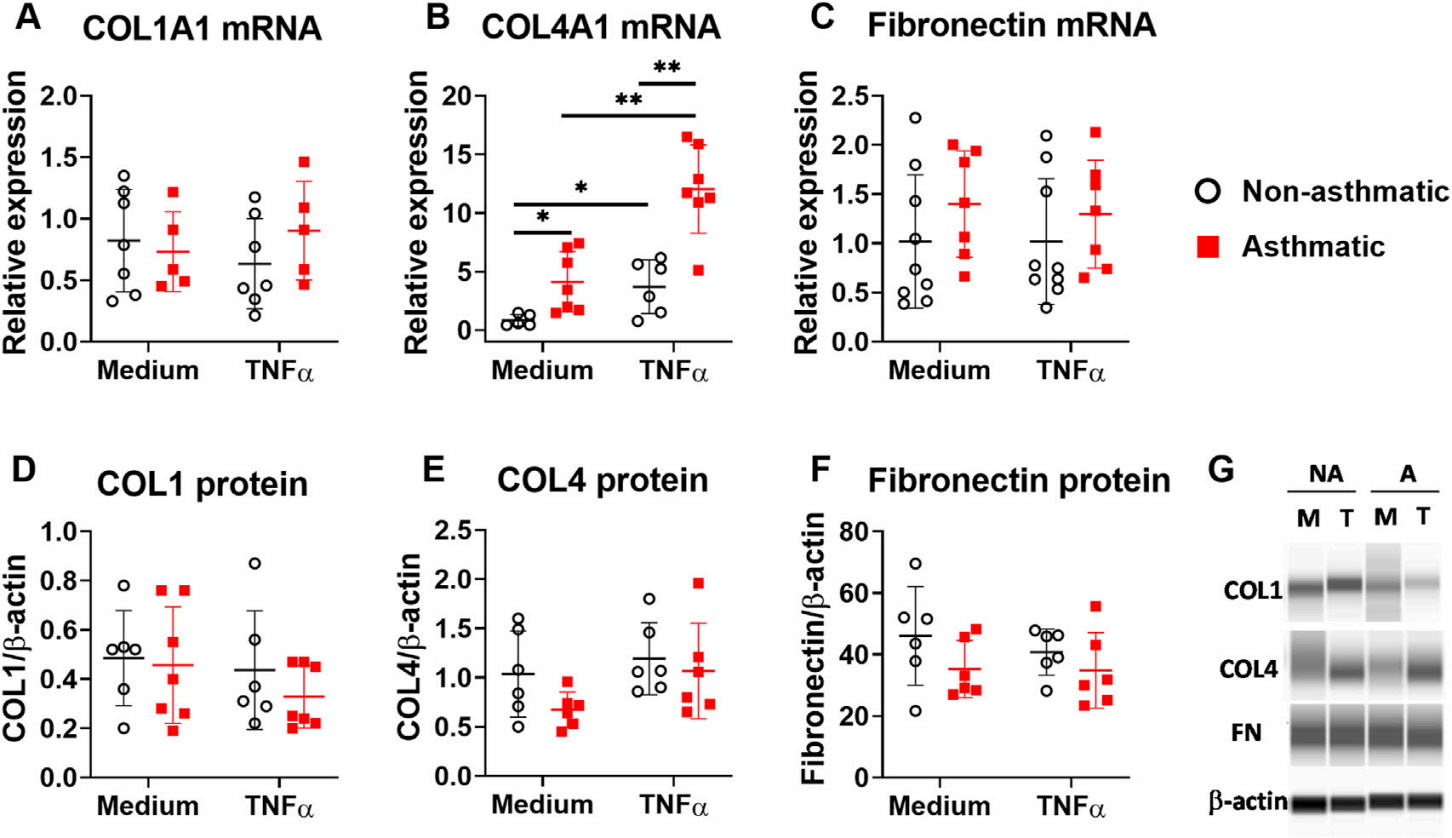
ECM expression in asthmatic and non-asthmatic human lung fibroblasts. Serum-starved fibroblasts were cultured with medium or TNFα (20 ng/ml) for 24 h or 3 days. RNA was extracted from the cells cultured for 24 h. Cell lysates were made after 3-day culture. Gene expression was determined by quantitative PCR **(A–C)**. The levels of ECM protein in the cell lysates were determined by WES and normalized to the expression of β-actin **(D–F)**. Representative WES images are shown in **(G)** (NA: non-asthmatic. A: asthmatic. M: medium. T: TNFα). Normalized data are shown as mean ± SD from N = 5–7 patient samples. Each dot represents one patient sample. * indicates significant difference *p* < 0.05. ** indicates significant difference *p* < 0.01.

One of the important functions of fibroblasts is to produce extracellular matrix (ECM) proteins to mediate tissue remodeling. We investigated whether TNFα regulates ECM production in lung fibroblasts by examining the expression of collagen type I (COL1), collagen type IV (COL4), and fibronectin. In both asthmatic and non-asthmatic fibroblasts, TNFα treatment did not induce significant changes of COL1 alpha 1 (COL1A1) and fibronectin mRNA expression (Figures 2A,C), although TNFα significantly upregulated the mRNA expression of COL4 alpha 1 (COL4A1) in these cells (Figure 2B). Compared to non-asthmatic controls, asthmatic fibroblasts had significantly higher levels of COL4A1 mRNA expression both at basal levels and after TNFα treatment. When protein expression was analyzed, TNFα treatment did not induce significant changes in COL1, COL4A1, and fibronectin in both non-asthmatic fibroblasts and asthmatic fibroblasts (Figures 2D–F). The protein expression levels for these ECM molecules were not significantly different between asthmatics and controls. Together these data suggest that TNFα preferentially stimulates proinflammatory cytokine responses but not ECM production in human lung fibroblasts.

### 3.3 Fibroblast-derived TSLP promotes type 2 cytokine production by PBMCs

**FIGURE 3:**
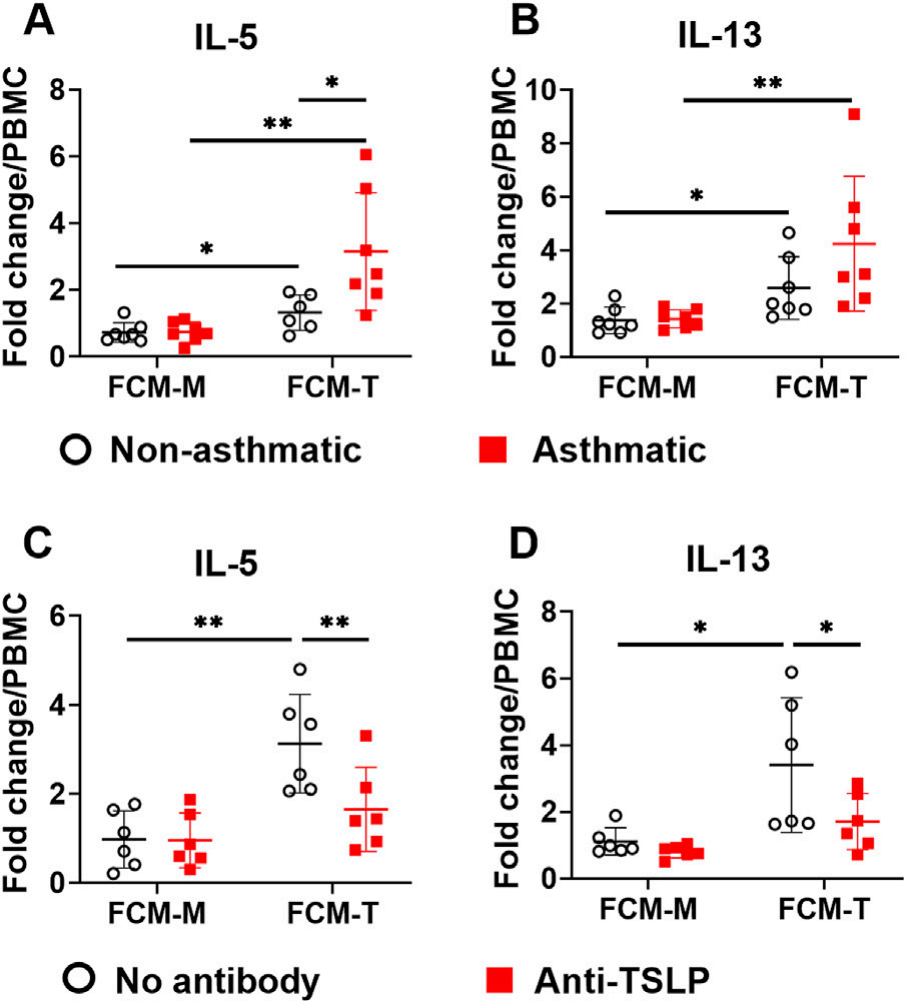
The effects of fibroblast conditioned medium (FCM) on IL-5 and IL-13 production by PBMCs. FCM was collected from either non-asthmatic or asthmatic lung fibroblasts that were cultured with medium (FCM-M) or TNFα (FCM-T) for 3 days. PBMCs were cultured with IL-33 (50 ng/ml) plus IL-2 (20 units/ml) with FCM-M or FCM-T for 4 days. IL-5 and IL-13 levels in the PBMC culture supernatants were measured by ELISA. Fold changes were quantified against the average value of PBMCs cultured with IL-33/IL-2 without FCM **(A,B)**. Anti-TSLP antibody (1 μg/ml) was added into the PBMC culture with FCM from asthmatic fibroblasts **(C,D)**. Fold changes were quantified against the average value of PBMCs cultured with IL-33/IL-2 without FCM. Data are shown as mean ± SD from N = 5–8 patient samples. Each dot represents one patient sample. * indicates significant difference *p* < 0.05. ** indicates significant difference *p* < 0.01.

TSLP, IL-33, and IL-25 are considered as “alarmins” that are secreted by lung epithelial cells upon airway exposure to pathogens or environmental insults (Hammad and Lambrecht, 2015). These cytokines play important roles in type 2 immunity and asthma pathogenesis (Corren and Ziegler, 2019). TSLP drives type 2 inflammation by activating multiple immune cell types, including dendritic cells, T lymphocytes, mast cells, innate lymphoid cells (ILCs), and macrophages. Since we observed that asthmatic fibroblasts secreted higher levels of TSLP (Figure 1H), we tested the hypothesis that TNFα-activated lung fibroblasts promote type 2 immune responses through TSLP pathway. To this end, we incubated healthy human PBMCs with 10% volume of fibroblast conditioned medium (FCM) that contained 0–800 pg/ml TSLP depending on the treatments (Figure 1H), and then examined the production of type 2 cytokines IL-5 and IL-13. FCM was collected from fibroblasts either cultured with medium (FCM-M) or with TNFα (FCM-T). Culturing PBMCs with either FCM-M or FCM-T did not induce IL-5 or IL-13 production that was measurable by ELISA (data not shown), suggesting that FCM alone was not sufficient to induce type 2 cytokine production in PBMCs. IL-33 has been shown to be a potent inducer for type 2 cytokine production by PBMCs (Bartemes et al., 2012). FCM-M and FCM-T did not have detectable levels of IL-33 by ELISA (data not shown). Since TSLP works synergistically with IL-33 (Mjosberg et al., 2012), we tested whether TSLP in FCM has synergistic effects with recombinant IL-33 in inducing type 2 cytokine responses in PBMCs. It has shown previously that IL-33 alone induces minimal amounts of IL-5 and IL-13, but IL-33 together with IL-2 induces significant production of IL-5 and IL-13 (Bartemes et al., 2014). Thus, we stimulated PBMCs with recombinant IL-33 plus IL-2 in the presence or absence of FCM and then measured IL-5 and IL-13 production. When FCM-M from either non-asthmatic or asthmatic fibroblasts was added into the PBMC culture, it did not have significant effect on IL-5 and IL-13 production (Figures 3A,B). In contrast, FCM-T from both non-asthmatic and asthmatic fibroblasts significantly enhanced IL-5 and IL-13 production. Importantly, FCM-T from asthmatic cells stimulated higher levels of type 2 cytokine production compared with non-asthmatic controls. To determine whether TSLP present in the FCM-T was responsible for this enhanced type 2 cytokine production by PBMCs, we added anti-TSLP antibody into the PBMC culture. Addition of the antibody significantly inhibited IL-33-induced IL-5 and IL-13 production when PBMCs were cultured with FCM-T derived from asthmatic fibroblasts (Figures 3C,D).

### 3.4 The expression of ER stress/UPR gene and protein is dysregulated in asthmatic lung fibroblasts

**FIGURE 4:**
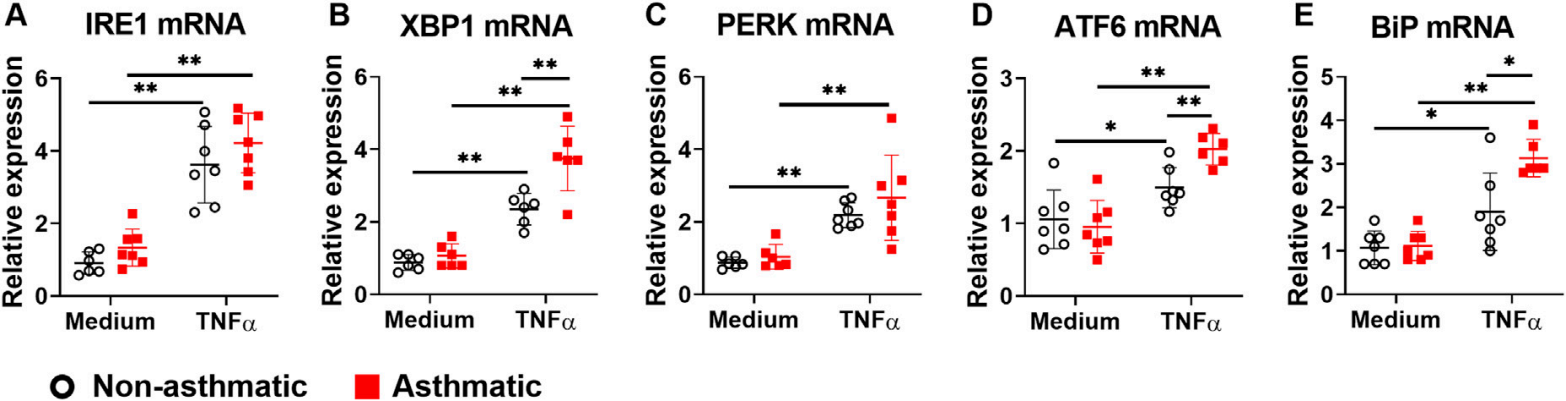
Gene expression of ER stress/UPR molecules in asthmatic and non-asthmatic human lung fibroblasts. Serum-starved fibroblasts were cultured with medium or TNFα (20 ng/ml) for 24 h. RNA was extracted from the cells. Gene expression was determined by quantitative PCR. Data are shown as mean ± SD from N = 5–7 patient samples. Each dot represents one patient sample. * indicates significant difference *p* < 0.05. ** indicates significant difference *p* < 0.01.

Since ER stress/UPR pathway is involved in asthma development (Miao et al., 2020) and TNFα activates this pathway in several cell types (Xue et al., 2005; Denis et al., 2010; Yap et al., 2020b; Zhao et al., 2021), we investigated whether TNFα activates ER stress/UPR pathway in primary human lung fibroblasts and whether asthmatic lung fibroblasts have dysregulated responses. First, we examined the mRNA expression of ER stress/UPR molecules including IRE1α and its downstream target XBP1, PERK, ATF6, and ER stress sensor chaperon BiP. At resting condition, none of these ER stress/UPR molecules showed significant differences in mRNA expression between asthmatic and non-asthmatic lung fibroblasts (Figure 4). TNFα treatment increased mRNA expression of all these molecules in both asthmatic and non-asthmatic lung fibroblasts. Moreover, TNFα-induced XBP1, ATF6, and BiP mRNA expression was significantly higher in asthmatics than in non-asthmatics, whereas IRE1α and PERK mRNA expression did not show significant differences between the two groups (Figure 4).

**FIGURE 5:**
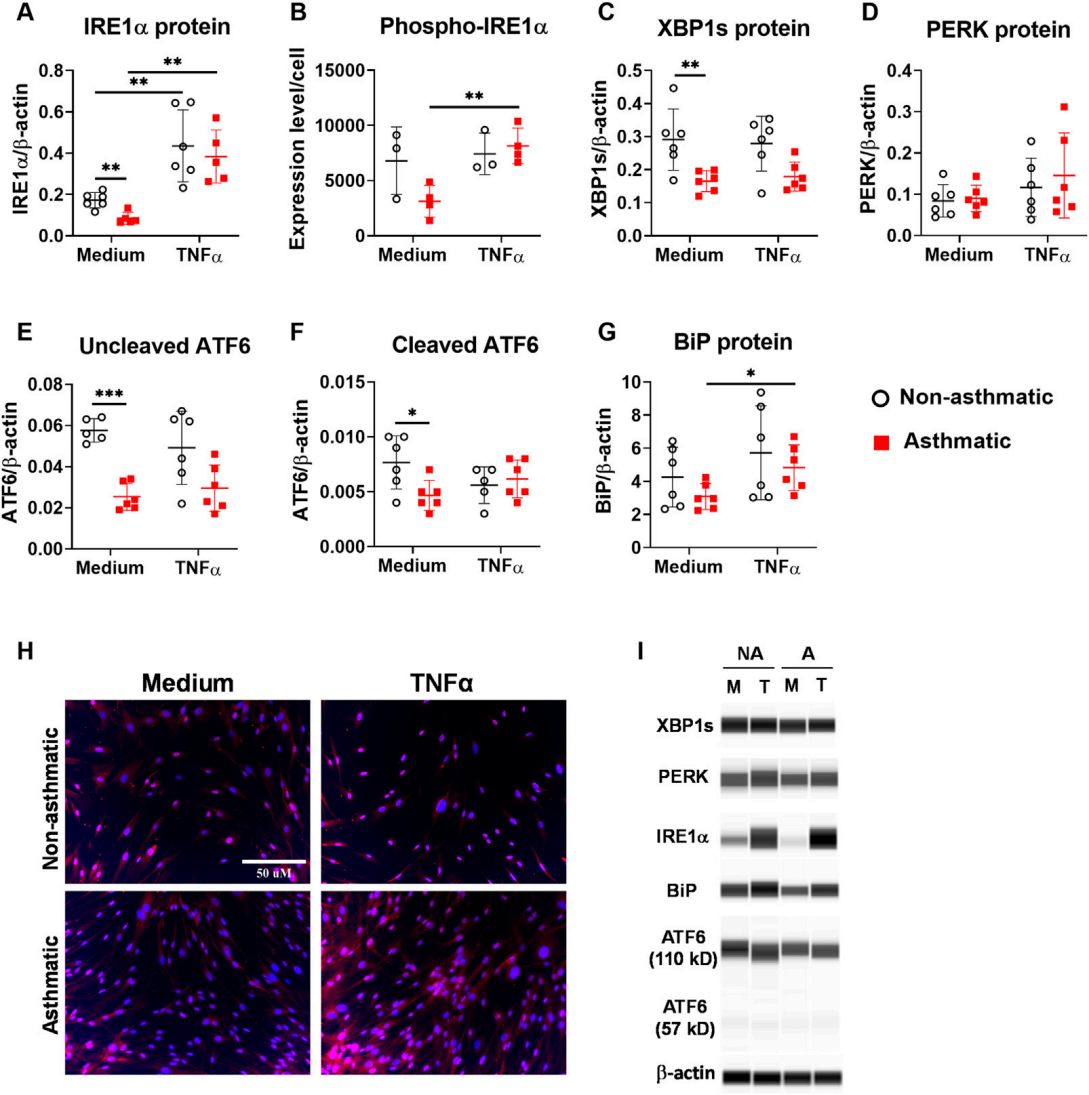
Protein expression of ER stress/UPR molecules in asthmatic and non-asthmatic human lung fibroblasts. Serum-starved fibroblasts were cultured with medium or TNFα (20 ng/ml) for 24 h. The protein expression in the cell lysates was determined by WES and normalized to the expression of β-actin **(A, C–G)**. Representative WES run images are shown in **(I)** (NA: non-asthmatic. A: asthmatic. M: medium. T: TNFα). Normalized protein expression data are shown as mean ± SD from N = 5–6 patient samples **(A, C–G)**. Phospho-IRE1α expression was quantified using images from immunofluorescence staining of fibroblasts and data are shown as mean ± SD from N = 3–4 patient samples **(B)**. Representative staining images are shown in **(H)**. Each dot represents one patient sample. * indicates significant difference *p* < 0.05. ** indicates significant difference *p* < 0.01.

Next, we analyzed the protein expression of the three major ER stress sensor pathways. First, we examined IRE1α and its downstream target XBP1. At resting condition, asthmatic fibroblasts had reduced expression levels of total IRE1α, phospho-IRE1α and XBP1s compared to non-asthmatic fibroblasts (Figures 5A–C). TNFα treatment significantly increased total IRE1α protein expression in both asthmatic and non-asthmatic cells, and the expression levels were similar between these two groups (Figure 5A). Since IRE1α is activated by phosphorylation, we examined whether TNFα induced the IRE1α phosphorylation in lung fibroblasts. Due to technical limitations of the WES system (incompatible, non-optimized antibodies) in detecting phopho-IRE1α, we used immunofluorescence staining for this particular readout. Interestingly, significant TNFα-induced IRE1α phosphorylation was observed in asthmatic fibroblasts but not in non-asthmatic cells (Figure 5B). As an IRE1α downstream target, XBP1 mRNA expression was significantly upregulated by TNFα (Figure 4B). However, TNFα treatment did not significantly change transcriptionally active XBP1s protein expression in either asthmatic or non-asthmatic fibroblasts (Figure 5C). Second, we examined the PERK sensor pathway. TNFα did not induce significant changes in PERK total protein expression in either asthmatic or non-asthmatic fibroblasts, and the expression levels were similar between the two groups (Figure 5D). Third, we examined ATF6 sensor pathway by measuring the expression levels of the inactive uncleaved ATF6 protein (about 110kD) and the active cleaved form of ATF6 protein (about 57kD). At resting condition, asthmatic fibroblasts had reduced expression levels of both uncleaved and cleaved ATF6 compared to non-asthmatic fibroblasts (Figures 5E,F). TNFα treatment slightly decreased both uncleaved and cleaved ATF6 expression in non-asthmatic fibroblasts. For asthmatic cells, TNFα did not induce significant changes in uncleaved ATF6 and slightly increased cleaved ATF6 expression. In addition to the three major ER stress sensor pathways, TNFα also significantly increased chaperone protein BiP expression in asthmatic fibroblasts while no significant effects were observed in non-asthmatic cells (Figure 5G). Altogether these data suggest that asthmatic lung fibroblasts have dysregulated ER stress/UPR pathway and IRE1α is predominantly affected in these cells.

### 3.5 Inhibition of ER stress/UPR attenuated TNFα-induced cytokine secretion

**FIGURE 6:**
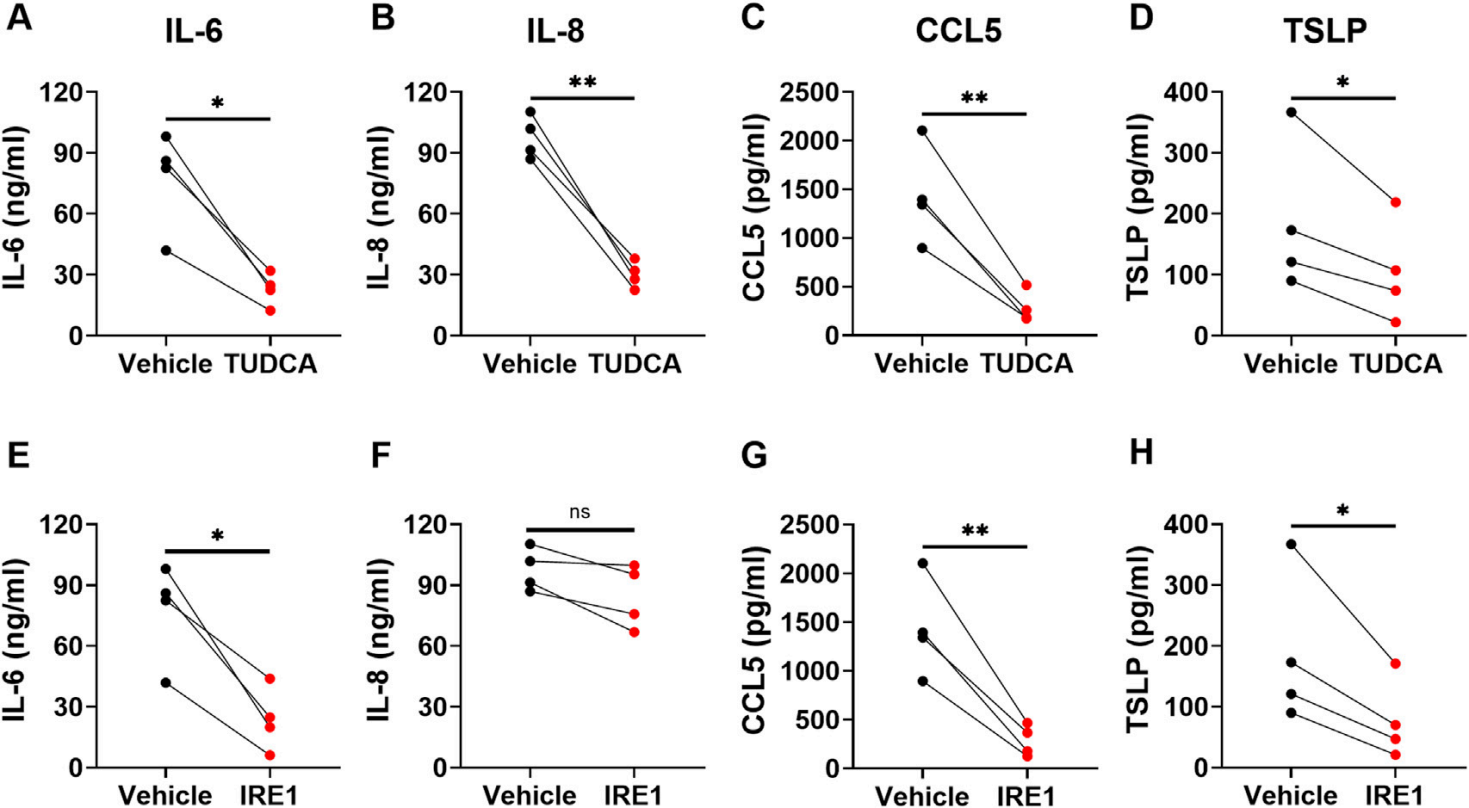
The effects of ER stress/UPR pathway inhibitors on cytokine/chemokine secretion by asthmatic lung fibroblasts. Asthmatic fibroblasts were pretreated with TUDCA at 500 μg/ml **(A–D)** or IRE1 inhibitor at 25 µM **(E–H)** for one hour and then cultured with TNFα (20 ng/ml) for 3 days. The levels of cytokines/chemokines in the culture supernatants were determined by ELISA. Data are shown as mean ± SD from N = 4 patient samples. Each dot represents one patient sample. ns: not significant. * indicates significant difference *p* < 0.05. ** indicates significant difference *p* < 0.01.

Studies suggest that ER stress pathway is involved in cytokine production (Smith, 2018). ER stress inhibitors have been shown to inhibit allergen-induced UPR and allergic airway disease in mice, suggesting a therapeutic potential for asthma treatment (Makhija et al., 2014; Siddesha et al., 2016; Nakada et al., 2019). To determine whether inhibition of ER stress/UPR pathway attenuates the production of cytokine/chemokine by asthmatic lung fibroblasts, we introduced small molecule inhibitors into the fibroblast culture, including TUDCA and IRE1 inhibitor I. TUDCA, a bile acid approved by US Food and Drug Administration for the treatment of certain cholestatic liver diseases, is commonly used for general ER stress/UPR inhibition. IRE1 inhibitor I specifically inhibits IRE1α endonuclease activity (Papandreou et al., 2011). Asthmatic lung fibroblasts were pre-treated with these inhibitors for 1 h before TNFα stimulation. Cytokine levels in the culture supernatants were measured 3 days after TNFα stimulation. By morphology and trypan blue staining, the ER stress inhibitors did not cause cell death. We found that TUDCA significantly inhibited TNFα-induced IL-6, IL-8, CCL5 and TSLP secretion (Figure 6). IRE1 inhibitor I significantly inhibited TNFα-induced IL-6, CCL5 and TSLP secretion, but only slightly inhibited IL-8 secretion. These data suggest that TNFα-induced IL-6, IL-8, CCL5 and TSLP secretion in asthmatic lung fibroblasts is differentially dependent on the activation of IRE1 pathway.

## 4 Discussion

In this study, we have investigated the proinflammatory cytokine responses in asthmatic and non-asthmatic human lung fibroblasts. Our results demonstrate that asthmatic lung fibroblasts contribute to asthmatic inflammation by secreting cytokines, such as TSLP, to drive type 2 immune responses. Mechanistically, TNFα, potentially derived from multiple cell types in the airway, stimulates proinflammatory cytokine secretion by lung fibroblasts, in part, through ER stress/UPR pathway.

While there is appropriate substantial focus on epithelium and immune cell derived inflammatory factors, lung fibroblasts can also contribute to airway inflammation and tissue remodeling by producing proinflammatory cytokines and ECM proteins (Ball et al., 2016; Hough et al., 2020). Although a number of proinflammatory mediators may contribute to asthma *per se*, and may even be derived from fibroblasts, in pilot studies using a multiplex assay, we found IL-6, IL-8, CCL5 and TSLP to be particularly expressed, and importantly changed by TNFα treatment. Accordingly, we focused on these four mediators. In response to TNFα, both asthmatic and non-asthmatic lung fibroblasts produced high levels of IL-6, IL-8, CCL5 and TSLP (Figure 1). However, we did not observe significant ECM protein upregulation under the same conditions (Figure 2). These data suggest that TNFα preferentially regulates cytokine responses in lung fibroblasts at least under the conditions of our experimental protocols. We found that asthmatic fibroblasts had increased levels of cytokines both at mRNA levels and secreted protein levels. However, the changes in mRNA expression did not always correlate with the changes in protein secretion from these cells. Increased IL-6 expression in asthmatic fibroblasts was seen at mRNA levels but not at protein levels. In contrast, CCL5 and TSLP were increased at protein levels but not at mRNA levels. These data indicate that complex multi-level mechanisms account for dysregulated cytokine responses in asthmatic lung fibroblasts. CCL5, a chemoattractant for many immune cells, including T cells, dendritic cells, eosinophils, natural killer cells, mast cells, and basophils, plays important roles in inflammation (Marques et al., 2013). We found that at basal levels, asthmatic lung fibroblasts secrete higher amounts of CCL5 compared to non-asthmatic cells. This increased CCL5 secretion may promote asthmatic inflammation by recruiting immune cells to lung parenchyma. TSLP is one of the epithelium-derived alarmins, plays important roles in type 2 immunity and asthma (Corren and Ziegler, 2019). Besides lung epithelial cells, fibroblasts are another cell source for TSLP production. Limited studies show that TSLP is produced in human nasal fibroblasts (Nomura et al., 2012) and mouse mesenchymal fibroblast-like lung cells (Dahlgren et al., 2019). Now we have shown that TSLP can also be produced and secreted by human lung fibroblasts. Importantly, asthmatic lung fibroblasts secreted more TSLP than non-asthmatic controls after TNFα stimulation. Furthermore, we have demonstrated that fibroblast-derived TSLP enhances IL-33-induced type 2 cytokine production by PBMCs. Altogether, our data suggest that increased TNFα expression in asthmatic lungs can further propel asthmatic inflammation through lung fibroblasts-TSLP-immune cells axis.

Accumulating studies reveal that the ER stress/UPR pathway plays important roles in asthma pathogenesis (Bradley et al., 2021). We have shown that at baseline, asthmatic lung fibroblasts had decreased protein expression for several ER stress molecules, including IRE1α, XBP1s, and ATF6, suggesting that the ER stress/UPR pathway is dysregulated in asthmatic lung fibroblasts. How asthma impacts ER stress/UPR pathway specifically in lung fibroblasts, and the likely different downstream effects of altered ER stress/UPR pathway are not known, and will form the basis of future studies. It has been shown that TNFα induces ER stress/UPR activation in murine fibrosarcoma L929 cells, human osteoblast-like SaOs-2 cells, synovial fibroblasts from patients with rheumatoid arthritis, and human airway smooth muscle cells (Xue et al., 2005; Connor et al., 2012; Yap et al., 2020b; Hameister et al., 2020). Adding to this information, our study has shown that TNFα induces ER stress/UPR activation in human lung fibroblasts. In non-asthmatic fibroblasts, TNFα stimulation significantly increased the mRNA expression of the three ER stress sensors, including IRE1α, PERK, and ATF6. TNFα also increased the mRNA expression of XBP1, a downstream target of IRE1α, and chaperone BiP. However, at protein levels, TNFα only induced significant upregulation of IRE1α but not others in fibroblasts. Moreover, TNFα increased the phosphorylation of IRE1α in asthmatic but not non-asthmatic fibroblasts. These data suggest that TNFα preferentially activates IRE1α pathway in lung fibroblasts, a finding similar to a previous report that TNFα selectively activated the IRE1α pathway but not the PERK and ATF6 pathways in human airway smooth muscle cells (Yap et al., 2020b). Although TNFα induced IRE1α activation and XBP1 mRNA upregulation in lung fibroblasts, TNFα did not induce significant changes in XBP1s protein expression, suggesting IRE1α may utilize other pathways, such as RIDD, to reduce ER stress in lung fibroblasts. Further studies are required to determine how TNFα regulates this pathway in lung fibroblasts.

Studies show that ER stress/UPR pathway is critically involved in inflammatory cytokine production. ER stress/UPR activation *per se* is sufficient to induce “sterile” inflammatory cytokine production. For example, pharmacologic ER stress inducers, such as tunicamycin, induced IL-1β, IL-6, and TNFα production in murine macrophages (Martinon et al., 2010; Kim et al., 2015). ER stress/UPR activation also regulates inflammatory stimulation-induced cytokine production. Treatment with pharmacologic ER stress inducers enhanced production of type I interferon (IFN), TNFα, IL-6, IL-1β, IFN-stimulated gene 15, and IL-23 by LPS stimulated macrophages and dendritic cells (Smith et al., 2008; Goodall et al., 2010; Martinon et al., 2010; Peters and Raghavan, 2011). In contrast, inhibition or deletion of ER stress/UPR molecules suppressed IL-4, IL-5, and IL-13 production in activated primary mouse CD4^+^ T cells (Kemp et al., 2013), decreased LPS-induced IL-1β, IL-6, and TNFα production in mouse macrophages (Martinon et al., 2010; Kim et al., 2015), and inhibited IL-23 production in human monocyte-derived dendritic cells (Goodall et al., 2010). In this study, we have found that both TUDCA, a general ER stress inhibitor, and an IRE1-specific small molecule inhibitor attenuated TNFα-induced IL-6, IL-8, CCL5, and TSLP production by asthmatic lung fibroblasts. IL-6, CCL5, and TSLP production was significantly inhibited by both inhibitors, suggesting that IRE1 activation plays a critical role in TNFα-induced IL-6, CCL5, and TSLP production/secretion by lung fibroblasts. IL-8 production was significantly inhibited by TUDCA but not by IRE1 inhibitor I, suggesting that IL-8 production/secretion is dependent on ER stress/UPR activation that may not require IRE1 endonuclease activity. Alternative mechanisms such as IRE1-mediated activation of NF-κB and activator protein 1 (AP-1) (Smith, 2018) might be involved in IL-8 production/secretion. We also observed that chaperone BiP protein expression was significantly increased by TNFα treatment in asthmatic lung fibroblasts. TUDCA has been shown to inhibit BiP expression (Uppala et al., 2017). Possibility exists that TUDCA worked through BiP to inhibit IL-8 production in our system.

In summary, we have shown that proinflammatory cytokine responses and ER stress/UPR pathway are dysregulated in asthmatic lung fibroblasts. TNFα, lung fibroblasts, and TSLP form an important axis to promote type 2 immune responses in asthmatic lungs that may contribute to the chronic inflammation in asthma. Proinflammatory cytokine responses in asthmatic human lung fibroblasts can be inhibited by pharmacologic inhibition of ER stress/UPR pathway, suggesting that ER stress/UPR pathway can potentially serve as a therapeutic target in asthma patients.

## Data Availability

The original contributions presented in the study are included in the article/supplementary materials, further inquiries can be directed to the corresponding author.

## Abbreviations

FCM, fibroblast conditioned medium; FCM-M, FCM from fibroblasts cultured with medium; FCM-T, FCM from fibroblasts cultured with TNFα.
