# Supplementary material for: Asthmatic lung fibroblasts promote type 2 immune responses via endoplasmic reticulum stress response dependent thymic stromal lymphopoietin secretion
Source: Front Physiol. 2023 Jan 25;14:1064822. doi: 10.3389/fphys.2023.1064822 (PMC9907026; doi:10.3389/fphys.2023.1064822)
Supplement: Supplementary file 1 [file Table1.DOCX]

**Supplemental Table 1. Clinical data of the fibroblast donors**

| **Patient** | **Sex** | **Age** | **Asthma severity** | **Lung function (FEV1)*** | **Asthma treatment** | **Ever smoked** |
| --- | --- | --- | --- | --- | --- | --- |
| **Control #1** | F | 68 | NA | Unknown | NA | No |
| **Control #2** | F | 69 | NA | Unknown | NA | Yes |
| **Control #3** | F | 67 | NA | Unknown | NA | Yes |
| **Control #4** | M | 65 | NA | Unknown | NA | Yes |
| **Control #5** | M | 67 | NA | Unknown | NA | Yes |
| **Control #6** | M | 68 | NA | Unknown | NA | Yes |
| **Control #7** | M | 67 | NA | Unknown | NA | Unknown |
| **Control #8** | M | 84 | NA | Unknown | NA | Yes |
| **Asthma #1** | F | 65 | Moderate | 69% | mometasone-formoterol albuterol | Unknown |
| **Asthma #2** | F | 70 | Moderate | 66% | budesonide-formoterol | Yes |
| **Asthma #3** | F | 73 | Moderate | 68% | fluticasone-salmeterol | Yes |
| **Asthma #4** | F | 66 | Moderate | 62% | budesonide | Unknown |
| **Asthma #5** | M | 62 | Moderate | 66% | unknown | Yes |
| **Asthma #6** | M | 79 | Unknown | 87% | unknown | Yes |
| **Asthma #7** | M | 67 | Moderate | 68% | fluticasone-salmeterol | No |

* No data on lung function (FEV1) for control donors were available in the medical records.
