# Supplementary material for: Asthmatic lung fibroblasts promote type 2 immune responses via endoplasmic reticulum stress response dependent thymic stromal lymphopoietin secretion
Source: Front Physiol. 2023 Jan 25;14:1064822. doi: 10.3389/fphys.2023.1064822 (PMC9907026; doi:10.3389/fphys.2023.1064822)
Supplement: Supplementary file 2 [file Table2.pdf]

Supplemental Table 2. Nucleotide sequence of PCR primers

Gene name	Forward	Reverse
s16	ATCAAGGTGAACGGGC	CAATGGTCTCATCAAGGTGAACGG
il6	GACCCAACCACAAATGCCAG	GTGCCCATGCTACATTTGCC
il8	CCTGATTCTGCAGCTCTGTGTGA	AATTTCTGTGTTGGCGCAGTGTGG
ccl5	AGTGTGTGCCAACCCAGAGAAGAA	TGTGGTAGAATCTGGGCCCTTCAA
col1a1	AAGACATCCCACCAATCACCTGCGTA	GCAGTTCTTGGTCTCGTCACAGATCA
col4a1	AAGGTGACAAAGGAAATCCAGGCTG	CGCCTTGATCACCTTTAATTCCCTGG
fibronectin	TGTCAGTCAAAGCAAGCCCGGTTGTT	CTCATAAGTGTCACCCACTCGGTAAG
xbp1	CCCTCCAGAACATCTCCCCAT	ACATGACTGGGTCCAAGTTGT
perk	AACCAGACGATGAGACAGAGTTGCGA	ACCTCTGGTTTGCTAAGGCTGGATGA
atf6	CACAGTGACGCTTCCTGAAAC	GCCATCATTAGGATCTGGGAGA
bip	CATCACGCCGTCCTATGTCG	CGTCAAAGACCGTGTTCTCG
ire1a	Custom designed by Integrated DNA Technologies	
